# Disease Interventions Can Interfere with One Another through Disease-Behaviour Interactions

**DOI:** 10.1371/journal.pcbi.1004291

**Published:** 2015-06-05

**Authors:** Michael A. Andrews, Chris T. Bauch

**Affiliations:** 1 Department of Mathematics and Statistics, University of Guelph, Guelph, Ontario, Canada; 2 Department of Applied Mathematics, University of Waterloo, Waterloo, Ontario, Canada; London School of Hygiene & Tropical Medicine, UNITED KINGDOM

## Abstract

Theoretical models of disease dynamics on networks can aid our understanding of how infectious diseases spread through a population. Models that incorporate decision-making mechanisms can furthermore capture how behaviour-driven aspects of transmission such as vaccination choices and the use of non-pharmaceutical interventions (NPIs) interact with disease dynamics. However, these two interventions are usually modelled separately. Here, we construct a simulation model of influenza transmission through a contact network, where individuals can choose whether to become vaccinated and/or practice NPIs. These decisions are based on previous experience with the disease, the current state of infection amongst one's contacts, and the personal and social impacts of the choices they make. We find that the interventions interfere with one another: because of negative feedback between intervention uptake and infection prevalence, it is difficult to simultaneously increase uptake of all interventions by changing utilities or perceived risks. However, on account of vaccine efficacy being higher than NPI efficacy, measures to expand NPI practice have only a small net impact on influenza incidence due to strongly mitigating feedback from vaccinating behaviour, whereas expanding vaccine uptake causes a significant net reduction in influenza incidence, despite the reduction of NPI practice in response. As a result, measures that support expansion of only vaccination (such as reducing vaccine cost), or measures that simultaneously support vaccination and NPIs (such as emphasizing harms of influenza infection, or satisfaction from preventing infection in others through both interventions) can significantly reduce influenza incidence, whereas measures that only support expansion of NPI practice (such as making hand sanitizers more available) have little net impact on influenza incidence. (However, measures that improve NPI efficacy may fare better.) We conclude that the impact of interference on programs relying on multiple interventions should be more carefully studied, for both influenza and other infectious diseases.

## Author Summary

The spread of infectious diseases can be inhibited by both vaccines and non-pharmaceutical interventions (NPIs) such as hand-washing, respiratory etiquette, and social distancing. Theoretical models of disease spread have incorporated how individuals make decisions concerning these interventions in the face of disease risks and intervention costs. However, most previous models have considered these two interventions separately from one another. Here we combine decision-making processes for both interventions in a single model that simulates influenza spread through a network. Individuals choose interventions based on their past and present experiences with influenza, and the personal and social impacts of their choices. Our model indicates that, due to feedback loops between the interventions via their mutual impact on disease levels, efforts to reduce influenza spread by expanding NPI practice are almost completely mitigated by the resulting drop in vaccine coverage, whereas efforts to expand vaccine coverage are only weakly affected by the response of NPI practice. Furthermore, strategies such as making vaccines more available, stressing the harms of being infected, or stressing the social benefits of preventing infection through both interventions will prevent more disease than only expanding NPI practice through making hand sanitizers more widely available, for example.

## Introduction

Infectious diseases continue to threaten human health throughout the world [1, 2]. In order to help alleviate these impacts, researchers have utilized mathematical models to improve our understanding of infectious disease dynamics [3]. In many cases, these models assume that human behaviour does not change over time or respond to disease dynamics in epidemiologically relevant ways. However, individual behaviour often does both influence–and evolve in response to–disease dynamics. For example, when vaccination is not mandatory, the prevalence of an infectious disease can depend on individual decisions of whether or not to vaccinate [4, 5]. Other behavioural practices that impact the spread of a disease include non-pharmaceutical interventions (NPIs) [6–10]. For susceptible individuals, NPIs can include hand washing or general avoidance of infectious individuals. For infectious individuals, these can include reducing contact with susceptible contacts, hand washing, or strict respiratory etiquette.

Mathematical models of the behavioral epidemiology of infectious diseases capture interplay between disease dynamics and individual behaviour [11] (we will refer to these as “disease-behaviour” models hereafter). These and similar types of models have focussed on vaccinating decisions where individual decision-making occurs according to a strategic environment or is determined by some other utility-based or rule-based mechanism [12–20]. Using such frameworks, Bauch [13], Fu et al. [15], Salathé and Bonhoeffer [20], and Reluga et al. [19] use models with imitation dynamics to predict potential vaccine uptake in populations. Similarly, Xia and Liu [21] base vaccination decisions not only on minimization of the associated costs, but also the impact that social influence has on each individual. d’Onofrio et al. [22] use an information dependent model where vaccination decisions are based on private and public information gathered about a disease. Further approaches by Vardavas et al. [23] incorporate memory of past disease prevalence, and Wells and Bauch [24] include memory of previous infections to study the effect of these factors on vaccinating behaviour. Other research has explored the impact of increased individual sexual risk behaviour on disease incidence, in response to the introduction of a hypothetical HIV/AIDS vaccine [25, 26], or how risk perception, HIV prevalence and sexual behaviour interact with one another in a core group population [27].

Other disease-behaviour models incorporate social distancing and other NPI-related behaviours. For example, Reluga [28] analyzes a differential game in which individuals choose a daily investment in social distancing in order to reduce the risk of infection. Funk et al. [29] allow information of a disease to spread over a network, and individuals protect themselves according to the quality of information they possess. Gross et al. [30] and Shaw and Schwartz [31, 32] study adaptive networks, where susceptible nodes rewire their connections from infectious to non-infectious nodes at a certain rate. Along the same vein, Zanette and Risau-Gusman [33] allow susceptible nodes to either permanently sever a connection with an infectious node, or rewire to another randomly chosen (and possibly infectious) node. Del Valle et al. [8] assume some individuals lower their contact rates once an epidemic is detected, whereas Glass et al. [34] and Kelso et al. [9] use complex contact networks which include families, schools, and workplaces to test differing social distancing methods such as school closures and the effects of staying at home while infectious.

Hence, disease-behaviour models studying either vaccinating behaviour or NPI behaviour separately from one another are relatively abundant, but models incorporating both types behaviour are rare, to our knowledge. However, for many infectious diseases (such as influenza) both NPIs and vaccines are part of infection control strategies, and both also respond to disease dynamics [4, 35]. Under these circumstances, it becomes important to study disease-behaviour interactions in populations where both vaccinating behaviour and NPI behaviour respond to disease dynamics. The effectiveness of one type of intervention may interfere with the effectiveness of the other intervention, through the mediator of disease dynamics. The objective of our research is to explore the interplay between individual decision-making (which is driven by methods from decision field theory [36]), regarding vaccines, NPIs, and disease dynamics in the context of influenza transmission and control, and to study the implications for disease mitigation strategies.

## Model

### Vaccination

We model the vaccination decision process using random walk subjective expected utility (SEU) theory, an intermediate stage in the mathematical derivation of decision field theory [36]. This approach allows us to capture the decision-making process of individuals in an uncertain environment. In the case of vaccination, the uncertainty lies in the chance of becoming infected in a given influenza season, depending on whether or not the individual is vaccinated and how effective the vaccine is.

**Fig 1:**
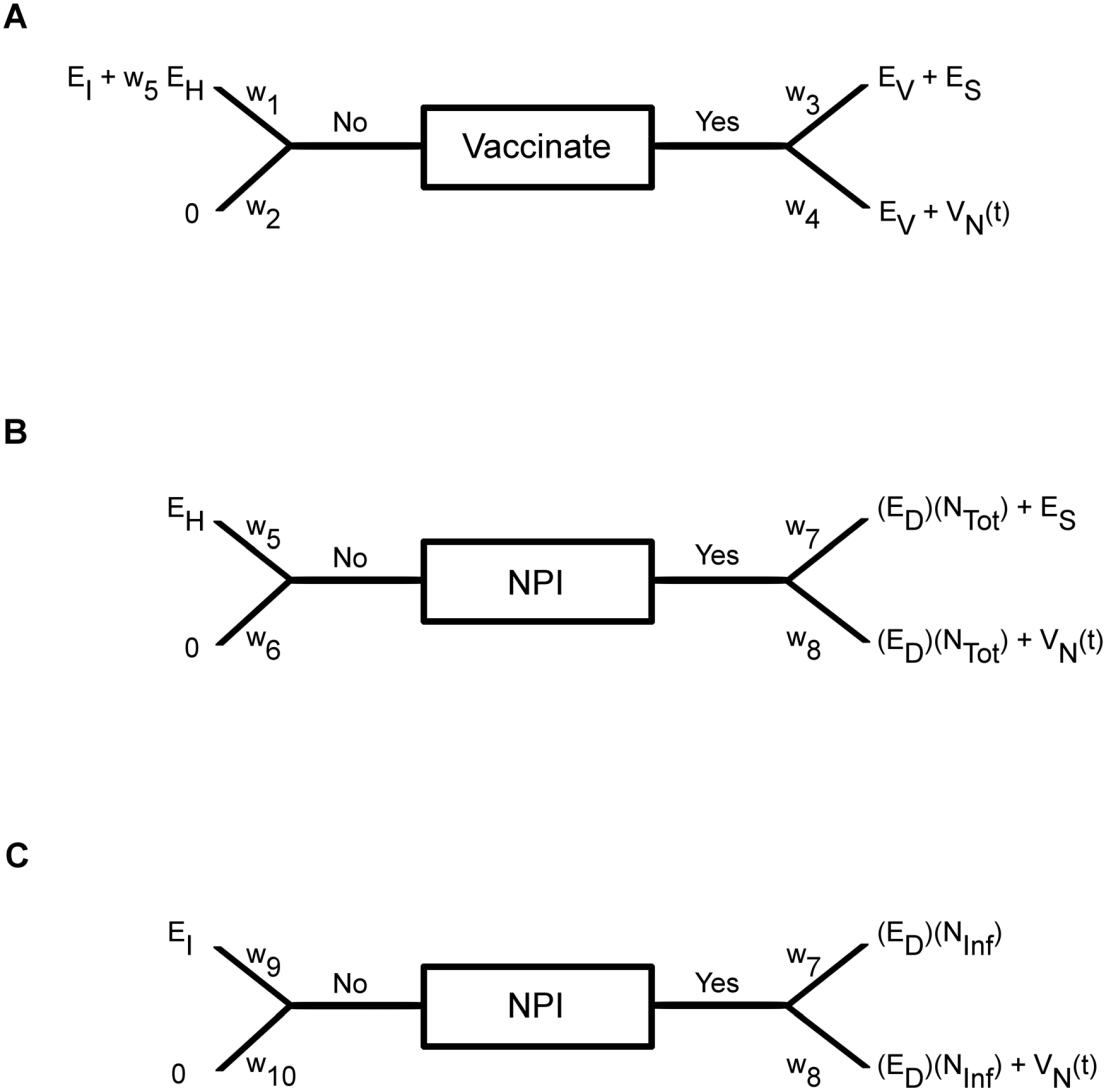
Vaccination and NPI decisions. (a) Diagram representing the vaccination choice problem. (b) Diagram representing the infectious NPI choice problem. (c) Diagram representing the susceptible NPI choice problem.

For the vaccinating decision, each susceptible individual compares the possible outcomes stemming from the “Yes” branch versus the possible outcomes stemming from the “No” branch (Fig 1a). The difference in these expected utilities, or ‘valence’ (*V*_*Y*_(*t*)−*V*_*N*_(*t*)), updates an individual’s preference, *P*(*t*), towards choosing one of these actions. The preference state of each individual is updated daily according to the rule:

$$\begin{array}{c} P\left(t\right)=P\left(t-1\right)+[V_{Y}\left(t\right)-V_{N}\left(t\right)]. \end{array}$$ (1)

If *P*(*t*) reaches a specified threshold, *θ*, then an individual decides to become vaccinated in that influenza season. Conversely, if *P*(*t*) reaches −*θ*, the individual decides not to become vaccinated that season. Intermediate values −*θ* < *P*(*t*) < *θ* can be interpreted as an individual being undecided regarding whether or not to vaccinate. If a choice is made, an individual’s preference state remains constant at *P*(*t*) = *θ* or *P*(*t*) = −*θ* until the beginning of the next season, when it then resets towards (1−*s*)*P*_*end*_ where *P*_*end*_ is the preference at the end of the last influenza season, and *s* is a memory decay factor.

Let us now define the social utility parameters associated with the vaccinating decision. The quantity *E*_*I*_ < 0 is the negative utility received (cost incurred) when an individual gets infected; *E*_*V*_ < 0 is the negative utility received (cost incurred) when an individual vaccinates; *E*_*S*_ > 0 is the positive utility received when an individual takes actions that they perceive will inhibit the spread of infection and therefore saves others from becoming infected; and *E*_*H*_ < 0 is the negative utility received (cost incurred) when an individual believes they are responsible for harming a neighbour by infecting them [37]. The baseline values for these and other parameters can be found in Table 1. If an individual chooses to not vaccinate during a season, they may become infected that season. If an individual instead chooses to vaccinate, the vaccine is effective for that season with probability *ϵ*_*V*_ (the vaccine efficacy) and otherwise the individual remains susceptible for the remainder of the season. The *w*_*i*_ parameters (0 ≤ *w*_*i*_ ≤ 1) represent the ‘subjective probability weights’ that determine the possible outcomes that are considered on a given day. In the case of vaccination, *w*_1_ is the probability an individual perceives of being infected if they do not vaccinate in a particular season. We assume *w*_1_ depends on how many of an individual’s neighbours have been infected in the current season, as well as their memory of the cumulative incidence from previous seasons:

$$\begin{array}{c} w_{1}=\sigma M_{H}\left(X_{n}\right)+\left(1-\sigma \right)\xi_{n-1}, \end{array}$$ (2)

where *σ* controls the relative importance of incidence from current versus past seasons, *X*_*n*_ is the cumulative number of neighbours who have become infected in the current influenza season *n*, *M*_*H*_(*x*) = 1−*e* ^−*κ*_*H*_*x*^ where *κ*_*H*_ is a proportionality constant controlling the perceived chance of becoming infected in a season, and *ξ*_*n*−1_ is an individual’s memory of incidence from past seasons:

$$\begin{array}{c} \xi_{n}=\sigma M_{H}\left(Y_{n}\right)+\left(1-\sigma \right)\xi_{n-1}, \end{array}$$ (3)

where *Y*_*n*_ is the cumulative number of an individual’s neighbours, including themselves, that have been infected by the end of season *n*. In this way, the memory of past influenza incidence declines with time according to *σ*.

**Table 1:** Model parameters with baseline values and sources.

**Parameter**	**Description**	**Value**	**Source**
*N*_*Pop*_	Number of Individuals in Network	10,000	[38–40]
*θ*_*V*_	Preference state threshold for vaccinating	0.1	Calibrated
*θ*_*D*_	Preference state threshold for distancing	0.1	Calibrated
*s*	Memory decay rate, per season	0.97	Calibrated
*σ*	Weight assigned to present state of infection in neighbours	0.5	Calibrated
*κ*_*H*_	Proportionality constant of perceived seasonal infection risk	0.3	Calibrated
*κ*_*C*_	Proportionality constant of perceived daily infection risk	0.05	Calibrated
*β*_0_	Average transmission rate	0.045	Calibrated
Δ*β*	Change in seasonality amplitude	0.2	[41]; Calibrated
*E*_*I*_	Cost incurred from becoming infected	−0.0055	[24]
*E*_*V*_	Cost incurred from vaccination	−0.0015	[24]
*E*_*D*_	Cost incurred from distancing a neighbour	−6 × 10^−6^	Calibrated
*E*_*S*_	Payoff for saving a neighbour from infection	6.5 × 10^−4^	Calibrated
*E*_*H*_	Cost incurred for infecting a neighbour(s)	−9 × 10^−4^	Calibrated
*ϵ*_*V*_	Vaccine efficacy	0.7	[42, 43]
*ϵ*_*NPI*_	NPI efficacy	0.4	[44, 45]
*ρ*	Probability of moving from state *R* to State *S*, per season	0.25	[46–48]
*ω*	Probability of moving from state *V* to State *S*, per season	0.5	[49]
*λ*	Average number of days to move from state *I* to state *R*	5	[46, 50–52]
*η*	Total number of exogenous infections per season	20	Calibrated

**Fig 2:**
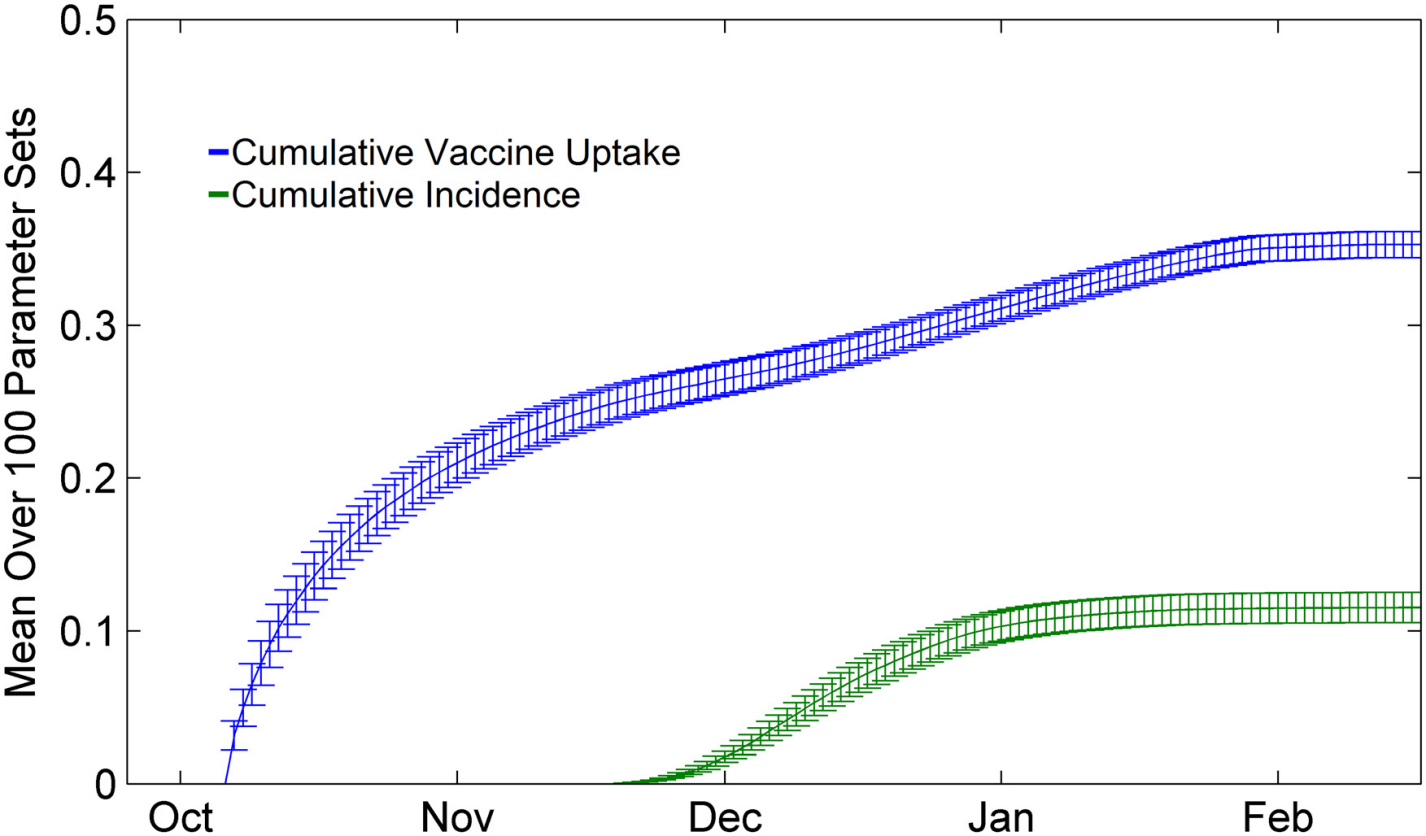
Seasonal time series. Example time series of vaccination (blue) and incidence (green) over a season. When a vaccine becomes available in early October, uptake increases in anticipation of the upcoming influenza season. Confidence intervals represent two standard deviations of outputs for the 100 parameter sets (see Model Calibration section).

Individuals may vaccinate during certain days of the year 280 < *t* or *t* < 40 (Fig 2), where *t* = 0 is January 1st, and the influenza season from the previous year is considered to end on day *t* = 285. We use this constraint because it reflects the typical timing of public influenza vaccination programs in fall and winter in many northern hemisphere countries, such as the United States and Canada. At the end of an influenza season, we set *Y*_*n*_ = *X*_*n*_ and then incorporate *Y*_*n*_ into the individual’s memory.

The second subjective probability weight is

$$\begin{array}{c} w_{5}=M_{C}\left(N_{susc}+\left(1-\epsilon_{V}\right)N_{vac}\right), \end{array}$$ (4)

where *M*_*C*_(*x*) = 1−*e* ^−*κ*_*C*_*x*^, *N*_*vac*_ and *N*_*susc*_ are the number of currently vaccinated and susceptible neighbours, respectively, and *κ*_*C*_ controls the perceived probability of infection. We interpret *w*_5_ as an individual’s perceived probability of infecting one or more neighbours, and the term *w*_5_ *E*_*H*_ captures the future outcome of an individual potentially infecting his/her neighbours that season after becoming infectious themselves, ultimately leading to a negative utility. To complete the outcomes of this branch, we note that the perceived probability of not becoming infected when choosing to not vaccinate is simply *w*_2_ = 1−*w*_1_. This outcome leads to a utility of 0.

On the ‘Yes’ branch, we define *w*_3_ = *ϵ*_*V*_ as the perceived probability that an individual is efficaciously vaccinated, thus *w*_4_ = 1 − *ϵ*_*V*_. In both cases, an individual knows that they must absorb the vaccine cost, *E*_*V*_. In the case of efficacious vaccinating, a positive utility *E*_*S*_ is also gained by protecting their neighbours for the remainder of the influenza season, which serves a similar function to the *w*_5_ *E*_*H*_ term stemming from the ‘No’ branch where an individual is considering future outcomes. In the case of inefficacious vaccination, an individual assumes that they may still become infected with a probability that increases with past and current disease incidence. This is represented by the term *V*_*N*_(*t*), the valence of the ‘No’ branch.

### Non-pharmaceutical Interventions

We model the NPI decision process using sequential SEU theory, a method similar to random walk SEU theory, but excludes the possibility that the preference state may start from a non-neutral initial value [36]. We use sequential SEU theory because we assume individuals make social distancing decisions on a day-to-day basis, dependent only on the current state of infection amongst their respective neighbours, whereas in the case of vaccination, the tendency to vaccinate or not can be carried over from one season to the next. Each infectious individual decides whether or not to practice NPIs to protect their neighbours for the duration of their illness (Fig 1b), and each susceptible individual decides whether to practice NPIs to protect themselves from their infectious neighbours that day (Fig 1c).

On the ‘Yes’ branch for the infectious NPI decision (Fig 1b), we have the probability of efficaciously using NPIs, *w*_7_ = *ϵ*_*NPI*_, or inefficaciously using them, *w*_8_ = 1−*ϵ*_*NPI*_. If NPIs are efficacious, they receive a positive utility *E*_*S*_ for saving susceptible neighbours from infection. However, if NPIs are inefficacious, they receive the valence *V*_*N*_(*t*) of the ‘No’ decision, on the branch associated with *w*_8_, representing that the outcome is the same as if they had never practiced NPIs to begin with. An individual believes that their use of interventions during their illness will be either fully effective or ineffective on all of their neighbours. Regardless of whether NPIs work or not, the infectious individual who practices NPIs pays a cost (*E*_*D*_)(*N*_*Tot*_) for having to utilize NPIs to protect their *N*_*Tot*_ neighbours, where *E*_*D*_ < 0 is the negative utility received (cost incurred) per neighbour. For the ‘No’ branch where the individuals decides not to practice NPIs, they may infect a neighbour with probability *w*_5_, receiving a negative utility (cost incurred) of *E*_*H*_ < 0 due to feeling responsible for spreading the infection. On the other hand, they infect no neighbours with probability *w*_6_ = 1−*w*_5_, thereby receiving a utility of zero.

NPI decisions are made by susceptible individuals who seek to protect themselves from their infectious neighbours in a similar way (Fig 1c). On the ‘Yes’ branch, an individual believes their NPIs will be efficacious with probability *w*_7_, receiving utility zero. If the NPIs are not efficacious, they receive the valence *V*_*N*_(*t*) from the ‘No’ branch. In either case, they pay a cost *E*_*D*_ in order to practice NPIs. On the ‘No’ branch, an individual who does not practice NPIs that day becomes infected with probability *w*_9_ = *M*_*C*_(*N*_*Inf*_), and receive a negative infection utility *E*_*I*_. With probability 1−*w*_9_, they believe they will not become infected and receive utility zero. We do not apply social utilities *E*_*H*_ and *E*_*S*_ in the susceptible NPI decision process because we assume as a first-order approximation that their decision focuses on short term outcomes (NPIs are only effective for the duration of infection, and may have to be repeated several times in the season, whereas a one-time vaccination decision will protect their neighbours from infection for the duration of the season). Also, if an individual is vaccinated, their perceived probability of becoming infected that day is reduced by 1−*ϵ*_*V*_. This reflects the fact that these individuals will believe themselves to have less chance of becoming infected than those who have not vaccinated. We note that infectious individuals practice NPIs on all of their neighbours, whereas susceptible individuals only practice them on their infectious neighbours. Infectious persons may stay home from work, and their hand washing benefits all susceptible persons with whom they come in contact with. In contrast, susceptible persons can be selective about avoiding infectious persons, or hand-washing after contact with infectious persons.

In our model, individuals are not aware of their own or their neighbours’ true susceptibility statuses. That is, they will make their intervention decisions based only on their own acquired knowledge which assumes everyone is susceptible at the beginning of each influenza season. This is in contrast to the true state of the system, which incorporates factors such as waning immunity. Moreover, the data we present on susceptible NPI rates only reports for those who are truly susceptible.

### Transmission Dynamics

The vaccination and NPI decision-making processes are embedded in an agent-based simulation model of influenza transmission through a static contact network. The contact network consists of 10,000 nodes through which influenza is transmitted, and was constructed by sampling a subnetwork from a larger contact network derived from census data from Portland, Oregon [38–40]. Previous research has confirmed that the subnetwork is a good approximation to the full network [24].

We assume a Susceptible-Infectious-Recovered-Vaccinated-Susceptible (*SIRVS*) natural history. Individuals move from the susceptible state *S* to the infectious state *I* with probability $Pr(t,N_{inf})=1-{\left(1-\beta (t)\right)}^{N_{inf}}$ per day, where *N*_*inf*_ is the number of infectious neighbours around the susceptible person on day *t*, and *β* is the transmission probability which varies seasonally according to $\beta (t)=\beta_{0}(1+\Delta \beta cos(\frac{2\pi t}{365}))$ [41]. If either the susceptible person or the infected person has opted to practice NPIs that day, then NPIs are effective with probability *ϵ*_*NPI*_, and that infected neighbour is not included in *N*_*inf*_ for the purposes of computing *Pr*(*t*, *N*_*inf*_). Infectious individuals recover (move from state *I* to state *R*) in a number of days sampled from a Poisson distribution with mean *λ* days. Individuals who have been efficaciously vaccinated with probability *ϵ*_*V*_ are moved to the *V* state, 14 days after being vaccinated [53]. Both recovered and vaccinated individuals become susceptible again at the beginning of each new season (day 285 of each year) with probabilities *ρ* and *ω*, respectively. In order to capture seasonal case importation, 5 randomly chosen susceptible individuals are made infectious every 10 days, from day 330 to 360.

Each day, the following sequence of events occurs: (1) each susceptible individual decides whether or not to practice NPIs on that day; (2) the following occur in a random order for each randomly chosen individual in the population: (i) If an individual is susceptible, they update their vaccination preference, (ii) if an individual is susceptible, they may become infected and make an infectious NPI decision, (iii) if an individual is infectious, they may recover.

### Model Calibration

To construct a baseline scenario, we calibrated the transmission probability *β*, amplitude of seasonality Δ*β*, and case importation rate *η* to the targets: (1) a cumulative seasonal incidence of approximately 15% to 20% in the absence of vaccination, and (2) infection prevalence that peaked, on average, between December and January of each year [24, 42, 54, 55]. These estimates come from North American (primarily, United States) populations. The calibrated value of Δ*β* was constrained on the interval (0.15, 0.3) [41].

We also calibrated the preference state threshold for vaccinating *θ*_*V*_, per season memory decay rate *s*, and proportionality constant for the perceived chance of becoming infected in an influenza season *κ*_*H*_ to the targets: (1) vaccine coverage of 30% to 40% per season, with (2) the majority of vaccinations occurring in October and November. These targets provide disease dynamics very similar to seasonal influenza. The utilities *E*_*I*_ and *E*_*V*_ were set according to Ref. [24], based in turn on Ref. [42, 50, 55–59]. Finally, the social utilities *E*_*D*_, *E*_*S*_, and *E*_*H*_ were calibrated to the targets: (1) an infectious individual practices NPIs with 87% probability, and (2) a susceptible individual practices NPIs with a 66% probability [60].

After obtaining this baseline scenario, we conducted three-point estimation Monte Carlo probabilistic sampling using triangular distributions to obtain sets of parameter values that yielded outputs within acceptable ranges. The triangular distributions were defined around the most uncertain baseline parameter values. Very broad ranges were used for the most uncertain parameters, to reduce model fitting issues due to having more parameters than calibration targets (generally, for each set of calibrated parameter values described above, there was one less target data point than the number of parameters to be calibrated) (Table 2). We repeatedly sampled parameter values from these distributions, and ran simulations using the sampled parameter sets. We discarded any parameter sets that yielded outcomes outside of a feasible range for vaccine uptake and NPI practice rates (Table 3). We accepted a larger range of NPI practice rates than for vaccine uptake, due to the greater uncertainties about the frequency of NPI practice for seasonal influenza [60]. In total, 2250 simulations were tested, providing a target number of 100 parameter sets yielding feasible outcomes. All simulations ran for 50 seasons with an initial population of susceptible individuals having no preference towards vaccinating and no perceived probabilities of becoming infected. For our results, the first 20 seasons of each simulation were discarded to remove transient effects.

**Table 2:** Sampling ranges for parameters used to obtain 100 baseline sets.

**Parameter**	**Sampling Limits**	**Sampling Mode**
*θ*_*V*_	[0.01, 0.2]	0.1
*θ*_*D*_	[0.01, 0.2]	0.1
*s*	[0.8, 0.99]	0.9
*σ*	[0.3, 0.7]	0.5
*κ*_*H*_	[0.1, 0.4]	0.25
*κ*_*C*_	[0.036, 0.054]	0.05
*E*_*D*_	[0.0, −12 × 10^−6^]	−6 × 10^−6^
*E*_*S*_	[0.0, 13 × 10^−4^]	6.5 × 10^−4^
*E*_*H*_	[0.0, −18 × 10^−4^]	−9 × 10^−4^

**Table 3:** Acceptance ranges for simulation averages across 30 seasons.

**Intervention Measure**	**Acceptance Range**
Vaccine Uptake	[30, 40]
Susceptible NPI Probability	[50, 75]
Infectious NPI Probability	[70, 90]

The data shows the average value per season over all 100 parameter sets, for vaccine coverage, infection incidence, probability of susceptible individuals practicing NPIs given that they encounter one or more infectious individuals on a given day (“susceptible NPI practice”), and probability of infectious individuals practicing NPIs while ill (“infectious NPI practice”).

## Results

### Baseline Scenario

**Fig 3:**
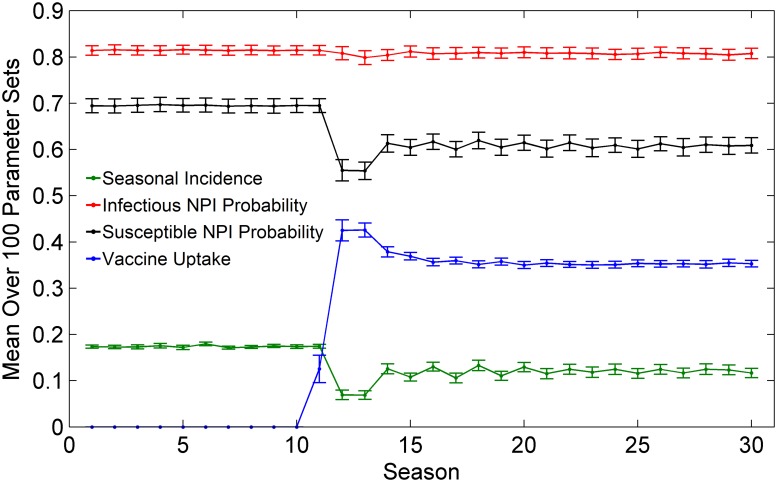
Impact of vaccine introduction. Example of a baseline scenario of our model where vaccination becomes available in season 10, causing a change in the vaccine coverage each season (blue), the seasonal infection incidence (green), the probability that a susceptible individual practices NPIs given that they encounter one or more infectious individuals on a given day (black), and the probability that an infectious individual practices NPIs while ill (red). Confidence intervals represent two standard deviations of outputs for the 100 parameter sets.

When vaccination is first introduced to the population, vaccine coverage climbs rapidly and peaks in the first few years after the vaccine becomes available, as members of the population adopt vaccination to avoid infection (Fig 3). As a result, seasonal influenza incidence decreases, which in turn causes a decrease in the probability that susceptible persons practice NPIs if they have an infected neighbour. This occurs because the reduced infection incidence due to the vaccine reduces the perceived infection risk among susceptible individuals, and thus makes them less willing to practice NPIs. After the initial peak in vaccine coverage and the corresponding dip in NPI practice, the vaccine uptake, infection incidence, and NPI practice rates equilibrate. In contrast to susceptible NPI practice, the infectious NPI practice rate stays almost constant during the whole period, due to the relative stability of the utilities found in the decision branches regarding this decision (Fig 1b). For example, an infectious individual deciding to use NPIs will always look to protect all of their neighbours, and this does not depend strongly on population-level incidence of infection that season. This is in contrast to a susceptible individual’s decision whether to use NPIs, which depends on how many of their neighbours are perceived to be infected.

### Interventions Can Interfere with One Another

**Fig 4:**
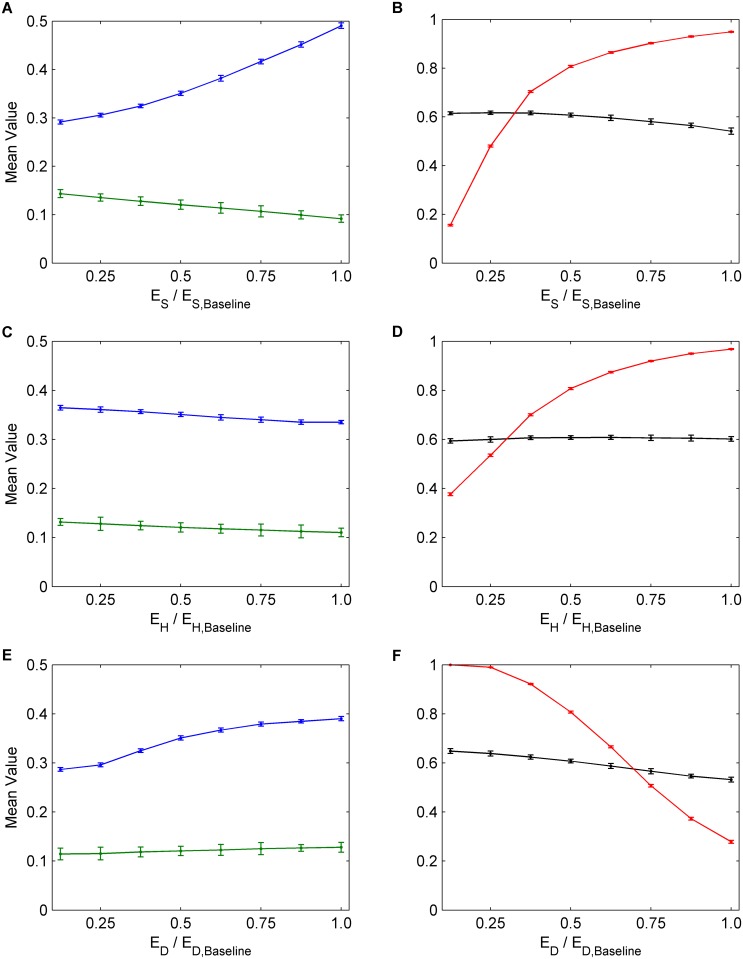
The effects of social parameters on interventions. Univariate sensitivity analysis for parameters *E*_*S*_, *E*_*H*_, and *E*_*D*_. The numbers on the horizontal axes correspond to multiples of the baseline values for *E*_*S*_, *E*_*H*_, and *E*_*D*_, hence 1.0 corresponds to the baseline value of the each parameter. (a), (c), (e) Average values across 30 seasons for vaccination coverage (blue) and incidence (green). (b), (d), (f) Average values across 30 seasons for NPI usage amongst susceptible individuals (black) and infectious individuals (red). Confidence intervals represent two standard deviations of the mean of the 100 parameter sets across 30 simulated seasons.

Next, we conducted a univariate sensitivity analysis, evaluating the impact of changes in baseline parameter values corresponding to measures that public health might take in order to improve outcomes. Increasing the utility for saving others from infection (*E*_*S*_) causes a significant reduction in infection incidence (Fig 4a and 4b). It also causes a slight decrease in NPI practice by susceptible individuals, but this is outweighed by large increases in both vaccine uptake and NPI practice by infectious persons that are sufficient to cause a net decline in infection incidence. These results illustrate a tradeoff whereby vaccine uptake, NPI practice among infectious individuals, and NPI practice among susceptible individuals cannot be simultaneously increased by changing *E*_*S*_. A reduced incidence due to expanding any one of these interventions will reduce the perceived infection risk and make individuals incrementally more complacent about preventing infection, which in turn reduces the uptake rates for the other interventions. Hence, each intervention tends to interfere with the other. Our focus in the remainder of this paper is to determine the conditions under which the interference between the two intervention types is strongest, which model parameters are most subject to interference, and how to bring about the greatest net reductions in infection incidence, despite interference. How interference plays out over time has already been described (Fig 3).

In contrast to significant reductions in infection incidence caused by increasing *E*_*S*_, increasing the cost for harming others (*E*_*H*_) causes only small net reductions in incidence, because a large increase in the NPI practice among infectious persons is strongly offset by a modest decline in vaccine uptake, while the rate of NPI practice by susceptible persons remains relatively constant (Fig 4c and 4d). Similarly, attempting to reduce incidence by decreasing the perceived cost of practicing NPIs (*E*_*D*_) also causes only a small net reduction in incidence, since the resulting increases in NPI practice among infectious and susceptible persons are again offset by reductions in vaccine uptake (Fig 4e and 4f).

**Fig 5:**
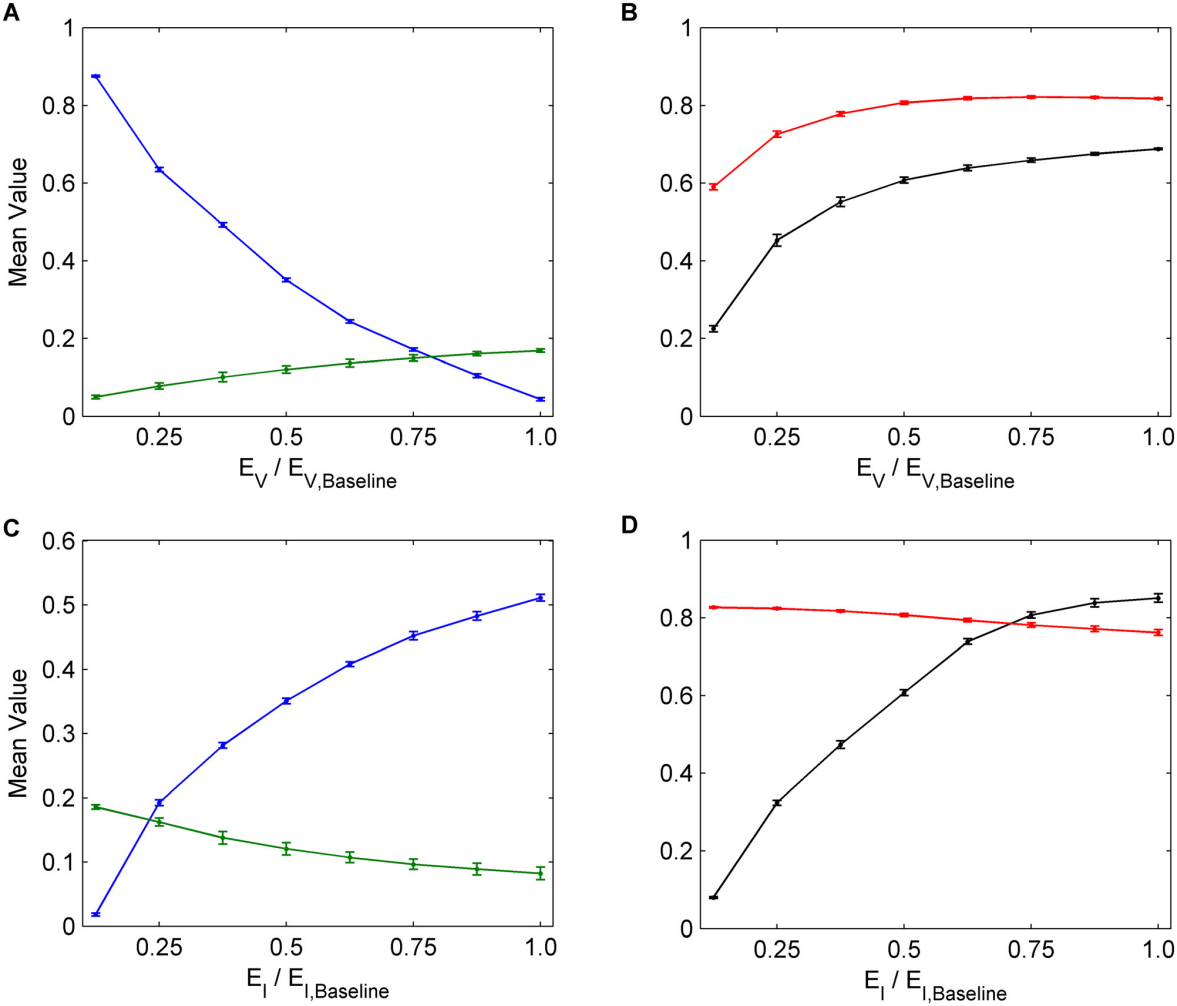
The effects of infection and vaccination costs on interventions. Univariate sensitivity analysis for *E*_*V*_ and *E*_*I*_. The numbers on the horizontal axes correspond to multiples of the baseline values for *E*_*V*_ and *E*_*I*_, hence 1.0 corresponds to the baseline value of the each parameter. (a), (c) Average values across 30 seasons for vaccination coverage (blue) and incidence (green). (b), (d) Average values across 30 seasons for NPI usage amongst susceptible individuals (black) and infectious individuals (red). Confidence intervals represent two standard deviations of the mean of the 100 parameter sets across 30 simulated seasons.

Decreasing the cost of vaccination (*E*_*V*_)–for instance by making the vaccine more easily accessible–results in significant reductions in infection incidence, because the significant increase in vaccine uptake is only partly offset by the resulting decline in NPI practice (Fig 5a and 5b). Likewise, increasing the perceived cost of infection (*E*_*I*_) causes an increase in both vaccine uptake and susceptible NPI practice, although infectious NPI practice remains relatively unchanged. The effect is a significant net decrease in incidence (Fig 5c and 5d). In summary, increasing the utility for saving others from infection (*E*_*S*_), decreasing the perceived cost of vaccination (*E*_*V*_), or increasing the perceived cost of infection (*E*_*I*_), are more effective in reducing infection incidence than changing perceived harms (*E*_*H*_) or perceived cost of NPI (*E*_*D*_), despite interference.

### Determining Which Interventions Interfere Most Strongly

In order to better understand how NPIs interfere with vaccine uptake, we compute the difference Δ*V* in vaccine uptake between the baseline scenario where individuals are free to practice susceptible and infectious NPIs versus a hypothetical scenario where they cannot practice either form of NPI. Similarly, to determine how vaccination interferes with susceptible (and infectious) NPI practice, we compute the difference Δ*N*_*S*_ in susceptible NPI practice rates (similarly, Δ*N*_*I*_ for infectious NPI practice rates) between the baseline scenario where individuals are free to choose vaccination versus a hypothetical scenario where vaccination is not available. We also compute the difference in seasonal incidence Δ*I* between the baseline scenario and all of these hypothetical scenarios.

#### Impact of interference on intervention uptake rates

**Fig 6:**
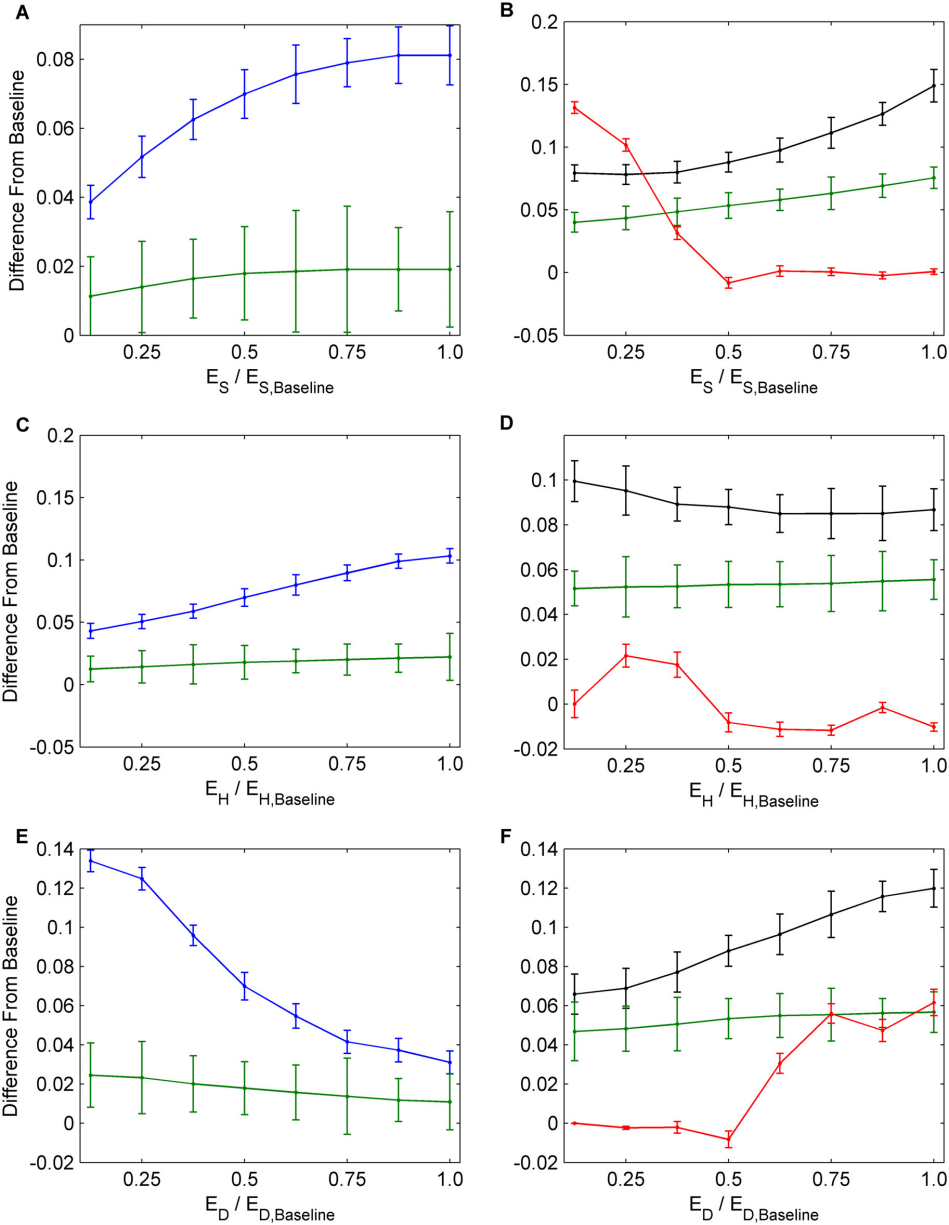
Interference between vaccination and NPIs. Univariate analysis for social parameters *E*_*S*_, *E*_*H*_, and *E*_*D*_ determining the amount that vaccination and NPIs interfere with each other in each scenario. (a), (c), (e) Average values across 30 seasons for change in vaccination coverage (blue) and change in incidence (green) between hypothetical scenarios without NPI usage and the baseline scenarios. (b), (d), (f) Average values across 30 seasons for change in NPI usage amongst susceptible (black) and infectious (red) individuals and change in incidence (green) between hypothetical scenarios without vaccine usage and the baseline scenarios. Confidence intervals represent two standard deviations of the mean of the 100 parameter sets across 30 simulated seasons.

**Fig 7:**
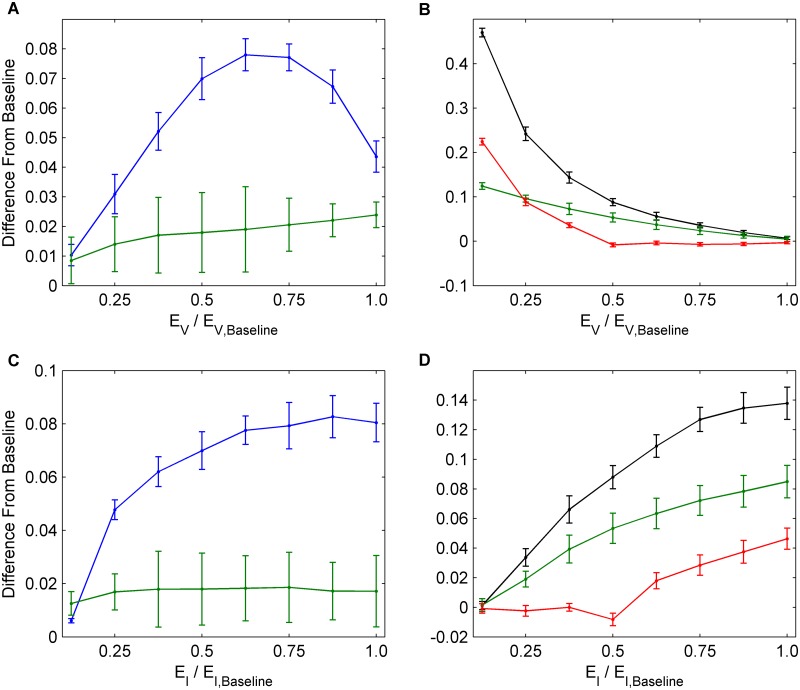
Interference between vaccination and NPIs. Univariate analysis for *E*_*V*_ and *E*_*I*_ determining the amount that vaccination and NPIs interfere with each other in each scenario. (a), (c) Average values across 30 seasons for change in vaccination coverage (blue) and change in incidence (green) between hypothetical scenarios without NPI usage and the baseline scenarios. (b), (d) Average values across 30 seasons for change in NPI usage amongst susceptible (black) and infectious (red) individuals and change in incidence (green) between hypothetical scenarios without vaccine usage and the baseline scenarios. Confidence intervals represent two standard deviations of the mean of the 100 parameter sets across 30 simulated seasons.

With respect to impacts of interference on intervention uptake rates, we observe that in general, across a broad range of utilities for *E*_*S*_, *E*_*H*_, *E*_*D*_, *E*_*V*_, and *E*_*I*_, NPI practice interferes significantly with vaccine uptake (Δ*V* usually ranging from 5% to 15%, Figs 6a, 6c, 6e, 7a and 7c). Vaccination also interferes significantly with susceptible NPI practice (Δ*N*_*S*_ usually ranging from 5% to 15%, Figs 6b, 6d, 6f, 7b, and 7d), but not as much with infectious NPI practice (Δ*N*_*I*_ is smaller, Figs 6b, 6d, 6f, 7b, and 7d). Infectious NPI practice is not as strongly affected because infectious NPI practice depends less on population prevalence than susceptible NPI practice does.

Interference of NPIs with vaccination is strongest at intermediate values of *E*_*V*_ (Fig 7a). When *E*_*V*_ is small, NPIs are not popular in the population and hence NPIs do not interfere strongly with vaccination. Thus, when NPIs are removed, there is little impact Δ*V* on vaccination levels. When *E*_*V*_ is large, vaccination becomes too costly for the population to adopt it widely. Therefore, vaccination levels do not increase by significant amounts when NPIs are added or removed from the population. When vaccination is not present, we see that for small *E*_*V*_, NPIs are interfered with especially strongly. This is because high vaccination coverage seen in the baseline scenario disappears, and thus individuals choose to practice NPIs more often. For larger values of *E*_*V*_, NPIs are undermined by decreasing amounts since vaccination levels in the baseline scenarios are greatly reduced (Fig 7b). Similar reasoning can explain trends in Δ*V*, Δ*N*_*I*_ and Δ*N*_*S*_ as other utilities are varied (Fig 7c and 7d).

#### Impact of interference on influenza incidence

Overall, when removing either vaccination or NPIs, we observe an increase in incidence, compared to baseline scenarios where both interventions are available (Δ*I* > 0; green lines in Figs 6 and 7). This occurs because having both interventions as an option is better than just having one of them.

However, the increase in incidence caused by removing vaccination (Figs 6b, 6d, 6f, 7b, and 7d) is much larger than the increase in incidence caused by removing NPIs (≈ 5–10% versus ≈ 1–2% Figs 6a, 6c, 6e, 7a, and 7c). Hence, vaccination appears to be a much stronger determinant of influenza incidence than NPI practice. When NPIs are introduced to the population, vaccine uptake declines sufficiently such that the net change in incidence Δ*I* is small, thus vaccination responds to the introduction of NPIs in a way that strongly mitigates the effectiveness of NPIs in reducing incidence (Figs 6a, 6c, 6e, 7a, and 7c). However, when vaccination is introduced to the population, the resulting decline in NPI practice is not sufficient to prevent significant changes in Δ*I* (Figs 6b, 6d, 6f, 7b, and 7d), thus NPIs do not strongly mitigate the effectiveness of vaccination. In other words, vaccination interferes strongly with the effectiveness of NPI practice, whereas NPI practice interferes weakly with the effectiveness of vaccination.

As a result of interference, efforts to boost NPI practice should be partially counter-productive due to the mitigating response of vaccine uptake, whereas efforts to boost vaccine uptake should be productive because the mitigating responses of NPI practice are not strong enough to prevent a significant decrease in influenza incidence. This trend is consistent across a broad range of parameter values.

### Understanding What Drives Different Levels of Interference for Different Interventions

**Fig 8:**
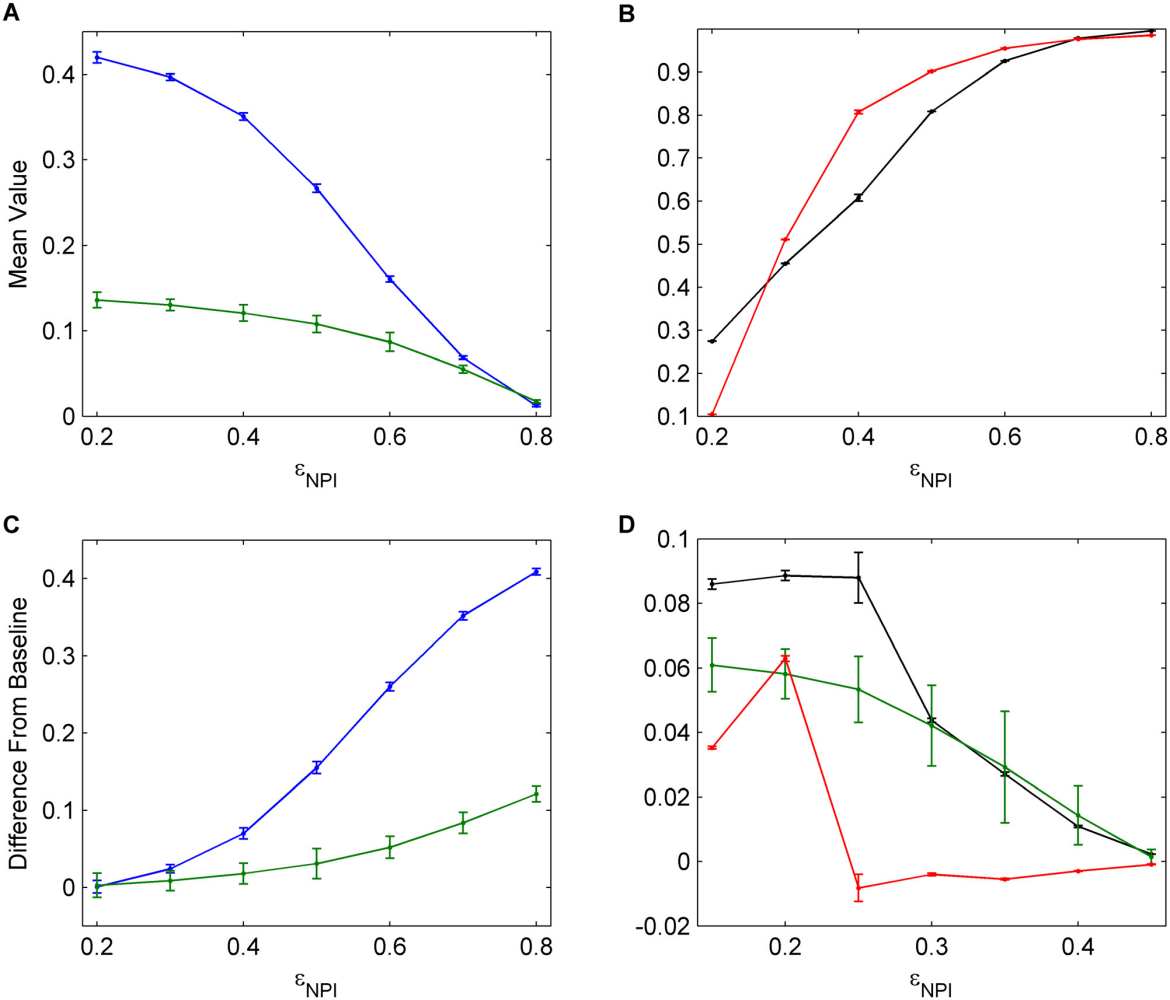
The effects of NPI efficacy. (a), (b) Univariate sensitivity analysis for NPI efficacy, *ϵ*_*NPI*_. Data shows average values across 30 seasons for vaccination coverage (blue), incidence (green), NPI usage amongst susceptible individuals (black), and NPI usage amongst infectious individuals (red). (c), (d) Determining the amount that vaccination and NPIs interfere with each other for various NPI efficacies. Average values across 30 seasons for change in vaccination coverage (blue), change in incidence (green), and change in NPI usage amongst susceptible (black) and infectious (red) individuals are shown. Confidence intervals represent two standard deviations of the mean of the 100 parameter sets across 30 simulated seasons.

**Fig 9:**
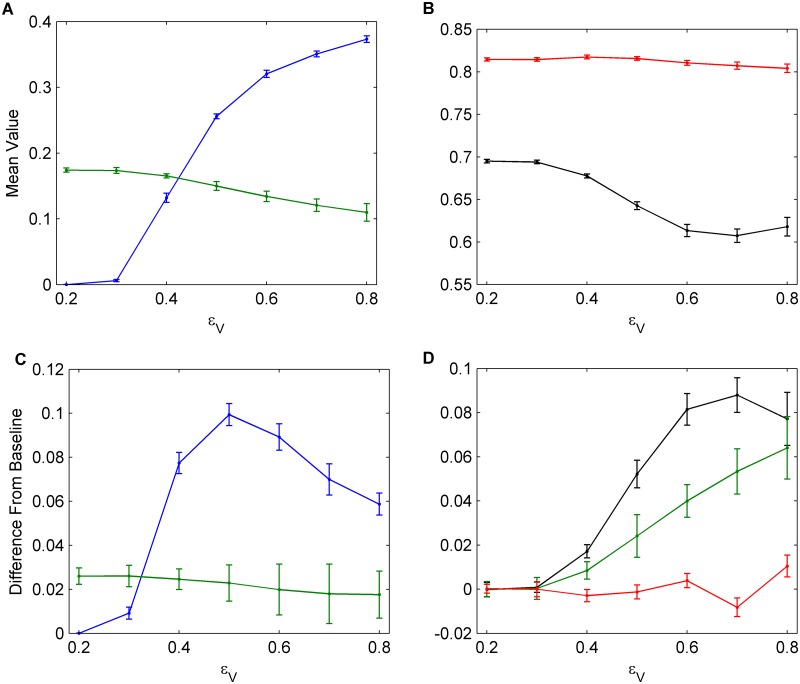
The effects of vaccine efficacy. (a), (b) Univariate sensitivity analysis for vaccine efficacy, *ϵ*_*V*_. Data shows average values across 30 seasons for vaccination coverage (blue), incidence (green), NPI usage amongst susceptible individuals (black), and NPI usage amongst infectious individuals (red). (c), (d) Determining the amount that vaccination and NPIs interfere with each other for various vaccine efficacies. Average values across 30 seasons for change in vaccination coverage (blue), change in incidence (green), and change in NPI usage amongst susceptible (black) and infectious (red) individuals are shown. Confidence intervals represent two standard deviations of the mean of the 100 parameter sets across 30 simulated seasons.

To understand the source of this asymmetry between the two interventions, we vary the NPI efficacy (*ϵ*_*NPI*_) and the vaccine efficacy (*ϵ*_*V*_) (Figs 8 and 9). As the NPI efficacy increases, the proportion of individuals practicing NPIs increases significantly (Fig 8b) and the vaccine uptake decreases in response, while the infection incidence also declines (Fig 8a). Similarly, when the NPI efficacy is very high, removing NPIs has a much larger impact on incidence than removing vaccination, the latter of which has almost no effect. But when the NPI efficacy is very low, the situation is reversed, and removing vaccination has a much bigger impact on incidence than removing NPIs (Fig 8c and 8d). These results show that feedback between interventions operates such that, if a less efficacious intervention is removed, the resulting increased uptake of the more efficacious intervention is sufficient to prevent a net increase in incidence. In contrast, if a more efficacious intervention is removed, the resulting increased uptake of the less efficacious intervention is not adequate to prevent an increase in incidence. Similar patterns hold when the vaccine efficacy (*ϵ*_*V*_) is varied, for similar underlying reasons (Fig 9). However, a secondary factor working in favour of vaccination is that vaccination–if efficacious–protects individuals throughout the influenza season, whereas NPI practice needs to be efficacious every time there is an infection in a network neighbour, in order for an individual to avoid infection throughout the entire season.

The difference in vaccine uptake with and without NPIs is highest for intermediate values of *ϵ*_*V*_. In general, this occurs because when intervention efficacy is very low, individuals will not adopt it, regardless of whether there is an alternative or not. Therefore, even if the alternative intervention is removed, individuals will tend not to increase adoption of the inefficacious intervention (Fig 9c, lowest values of *ϵ*_*V*_). On the other hand, if an intervention is significantly more effective than the alternative intervention, or not very costly, then it will continue to be used by a large proportion of the population, and will not experience significant interference from the less effective alternative intervention which does not significantly change incidence (Fig 9c, highest values of *ϵ*_*V*_).

In summary, these results show that, the more efficacious an intervention is, the less its effectiveness will be compromised by the other intervention, but the more it will compromise the effectiveness of other intervention. The central role of intervention efficacy also explains why the highest reductions in incidence occur when utilities that support vaccination only are changed (e.g. the vaccine cost is reduced, Fig 5a and 5b), or when utilities that support both vaccination and NPI practice are changed (e.g. when the payoff for saving others from infection is increased, Fig 4a and 4b, or the perceived cost of infection is increased, Fig 5c and 5d). In contrast, decreasing the perceived cost of social distancing *E*_*D*_ has little impact on incidence (Fig 4e and 4f), since this NPI practice is interfered with by the mitigating response of vaccine uptake.

## Discussion

We have constructed a seasonal influenza transmission model that incorporates how behavioural decisions for both individual vaccinating decisions and individual NPI practice (hand-washing, social distancing) respond to changes in infection incidence. Our population was distributed across an empirically-based network, and parameter values were based either on literature [24, 41–52, 55], or were calibrated to typical influenza seasonal patterns using a probabilistic sampling approach.

These results illustrate how vaccine uptake and NPI practice interfere with one another. If vaccine coverage increases, the resulting change in transmission patterns causes a decrease in the practice of NPIs. This is especially true for susceptible individuals, since susceptible NPI practice is more sensitive to population incidence. Similarly, if NPI practice expands, vaccine coverage will decrease by a roughly similar amount.

Although susceptible NPI practice and vaccine coverage have similar impacts on each other’s uptake, the impact on incidence is highly asymmetrical between the two interventions: the effectiveness of NPI practice is strongly mitigated by the response of vaccine uptake, whereas the effectiveness of vaccination is only weakly mitigated by the response of NPI practice. This asymmetry is driven by the differing efficacies of the two types of interventions: the higher the efficacy of the intervention (*ϵ*_*V*_, *ϵ*_*NPI*_), the less its effectiveness in terms of reducing influenza incidence will be mitigated by the other intervention, but the more it will mitigate the effectiveness of other intervention.

Because influenza vaccine efficacy is generally higher than NPI efficacy, these effects have potentially important implications for influenza mitigation strategies. Efforts to boost NPI practice could be strongly counteracted by the resulting declines in vaccine coverage, hence boosting NPI practice could be counter-productive. However, boosting vaccine coverage can still be productive since the resulting response of NPI practice will not as strongly mitigate the effectiveness of expanded vaccine coverage. Because of the role of efficacy, increasing NPI efficacy through fostering better hand-washing techniques or respiratory etiquette might more useful than only increasing NPI uptake rates.

As a result of this asymmetry, we observed that increasing the utility for saving others from infection, *E*_*S*_ was the most effective way of decreasing incidence because it supports both vaccination and NPI practice. From the standpoint of an advertising campaign, this would mean highlighting the fact that saving friends and family from becoming ill, both through vaccination and through NPIs, would be effective. In contrast, attempting to expand NPI practice without simultaneously encouraging vaccine uptake (for example through making hand sanitizer stations more widely available) could be counter-productive since NPI efficacy is not as high as vaccine efficacy. Similarly, increasing the perceived cost of infection, *E*_*I*_ was also found to be an effective way to reduce incidence, since both vaccination and NPI practice are thereby supported.

The asymmetry also explains why decreasing the cost of vaccination, *E*_*V*_, was observed to be effective. The resulting expansion in vaccine uptake suppresses NPI practice to some extent, but because vaccine efficacy is higher than NPI efficacy, decreasing *E*_*V*_ still causes net reductions incidence. Hence, reducing the perceived cost of the vaccine by expanding availability (through more seasonal influenza vaccine clinics) or decreasing its price will reduce influenza incidence, despite interference.

Our model makes several simplifying assumptions with respect to decision making. Firstly, we group all NPIs into two categories: those utilized by susceptible individuals, and those utilized by infectious individuals. In reality, however, an infectious individual may practice combinations, such as choosing to practice strict respiratory etiquette, but not staying home to isolate themselves. Both forms of NPI likely have differing efficacy, hence our grouping assumption could influence results. Similarly, we used a common cost parameter, *E*_*D*_, for all NPIs, but different forms of NPI would likely impose varying costs. We also allowed our population to have knowledge of both vaccine and NPI efficacies.

Additional factors that could impact the decision making processes are misinterpretations of influenza-like illnesses (ILIs) as being cases of influenza. Often, individuals may mistake other respiratory illnesses for influenza, artificially inflating perceived infection numbers and impacting perceived vaccine efficacy. Similarly, we assume only a single strain of influenza, when in reality, there are often multiple circulating strains. The model could be improved in future research by adding further heterogeneity such as age structure, making perceived efficacy depend on individual experience with interventions, and introducing a probability of ILI being mistaken for influenza, or *vice versa* [24]. The contact network could also be modified to include family and work structures, which may in turn influence memories of previous infections and perceived infection risk. For example, individuals may weigh the fact that they have had a family member who was recently infected more so than if a casual contact like a co-worker recently fell ill. Moreover, individuals may be more inclined to practice NPIs around family members than their other contacts. Finally, personal infection history may be considered to be significantly more important than neighbour infection history when evaluating perceived risks, which is not accounted for in the model.

Being a highly parametrized model, there are several drawbacks associated with calibrating the model to empirical targets in order to obtain a baseline parameter set. We took parameter values directly from estimates in the available literature whenever possible, but (especially for NPIs) there is a dearth of information regarding intervention behaviour and impact for seasonal influenza [44], necessitating calibration. As a result, we had more calibrated parameters than calibration targets (see Methods), meaning that alternative baseline parameter sets could have matched the data almost as well as the baseline set that we used. Our adoption of very broad sampling intervals for the probabilistic uncertainty sampling partially addresses this since the broad intervals will include those parameter values, however, the resulting frequency distribution of outcomes could still vary depending on what baseline parameter set is used as the baseline for defining the triangular distributions. Future work could explore alternative parameterization methods, such as Latin hypercube sampling, which might help overcome this limitation.

In conclusion, interference stemming from feedbacks between interventions and disease dynamics can comprise the realized effectiveness of those interventions for reducing influenza incidence, depending on the clinical efficacy of the interventions in individuals. Health authorities and epidemiologists should further explore the potential for interference between different interventions for the same infectious disease, and formulate infection control strategies accordingly.

## Supporting Information

S1 Text Java code used for simulations. (JAVA)

S2 Text Java code used for simulations. (JAVA)

S1 Data Parameter sets used for simulations. (TXT)

S2 Data Network used for simulations. (TXT)
